# Improving Prediction of Surgical Site Infection Risk with Multilevel Modeling

**DOI:** 10.1371/journal.pone.0095295

**Published:** 2014-05-16

**Authors:** Lauren Saunders, Marion Perennec-Olivier, Pascal Jarno, François L’Hériteau, Anne-Gaëlle Venier, Loïc Simon, Marine Giard, Jean-Michel Thiolet, Jean-François Viel, for the RAISIN group

**Affiliations:** 1 Coordination Centre for Nosocomial Infection Control, Western Regions, Rennes, France; 2 Department of Public Health, Faculty of Medicine, Rennes, France; 3 Coordination Centre for Nosocomial Infection Control, Northern Regions, Paris, France; 4 Coordination Centre for Nosocomial Infection Control, South-Western Regions, Bordeaux, France; 5 Coordination Centre for Nosocomial Infection Control, Eastern Regions, Nancy, France; 6 Coordination Centre for Nosocomial Infection Control, South-Eastern Regions, Lyon, France; 7 Institute for Public Health Surveillance, Saint Maurice, France; University of Pittsburgh, United States of America

## Abstract

**Background:**

Surgical site infection (SSI) surveillance is a key factor in the elaboration of strategies to reduce SSI occurrence and in providing surgeons with appropriate data feedback (risk indicators, clinical prediction rule).

**Aim:**

To improve the predictive performance of an individual-based SSI risk model by considering a multilevel hierarchical structure.

**Patients and Methods:**

Data were collected anonymously by the French SSI active surveillance system in 2011. An SSI diagnosis was made by the surgical teams and infection control practitioners following standardized criteria. A random 20% sample comprising 151 hospitals, 502 wards and 62280 patients was used. Three-level (patient, ward, hospital) hierarchical logistic regression models were initially performed. Parameters were estimated using the simulation-based Markov Chain Monte Carlo procedure.

**Results:**

A total of 623 SSI were diagnosed (1%). The hospital level was discarded from the analysis as it did not contribute to variability of SSI occurrence (p  = 0.32). Established individual risk factors (patient history, surgical procedure and hospitalization characteristics) were identified. A significant heterogeneity in SSI occurrence between wards was found (median odds ratio [MOR] 3.59, 95% credibility interval [CI] 3.03 to 4.33) after adjusting for patient-level variables. The effects of the follow-up duration varied between wards (p<10^−9^), with an increased heterogeneity when follow-up was <15 days (MOR 6.92, 95% CI 5.31 to 9.07]). The final two-level model significantly improved the discriminative accuracy compared to the single level reference model (p<10^−9^), with an area under the ROC curve of 0.84.

**Conclusion:**

This study sheds new light on the respective contribution of patient-, ward- and hospital-levels to SSI occurrence and demonstrates the significant impact of the ward level over and above risk factors present at patient level (i.e., independently from patient case-mix).

## Introduction

Surgical site infection (SSI) is one of the most frequent hospital-acquired infections occurring in surgical patients and leads to increased morbidity, mortality and costs [1]–[3].

Since the early 1980s, SSI surveillance which provides appropriate data feedback (risk indicators, clinical prediction rule, etc.) to surgeons is considered an important component of the strategies developed to reduce SSI occurrence [4]–[6].

In this context, many countries have developed a national system for the surveillance of nosocomial infections. In France, a national surgical site infection surveillance system (the RAISIN surveillance system) based on a pyramidal organization (local, regional and national) was implemented in 1999 [7], using standard guidelines established in 1992 by the American Centers for Disease Control and Prevention (CDC)’s National Nosocomial Infections Surveillance (NNIS) system [4]. These guidelines were recently updated by the French Technical Committee for Nosocomial Infections and Healthcare Associated Infections [8]. The RAISIN surveillance system relies on volunteer surgical wards from public or private hospitals that routinely collect nosocomial infection data. This surveillance system reported a crude incidence rate of one SSI per 100 procedures in 2009–2010 [9].

To improve the accuracy of surveillance, successive risk indicators have been suggested [10]–[12]. They are mainly based on stratification or adjustment of SSI risk such as the NNIS risk index (consisting of 4 categories based on the American Society of Anesthesiologists [ASA] physical status score, wound class and duration of surgery). The SSI incidence rate among patients with an NNIS risk index of 0 is now provided by many surveillance systems. A more recent approach uses the Standardized Infection Ratio (SIR), calculated as the number of reported SSI divided by the number of expected SSI for a given ward and year [13]. The latter is the sum of individual SSI probabilities calculated from a logistic regression reference model including only individual risk factors [9]. As reported by Rioux et al. [14], the SIR was found to be a more reliable indicator when estimating the reduction in SSI incidence than the NNIS-0 SSI incidence rate, as it includes individual characteristics. Thus, each ward participating in the RAISIN surveillance system currently uses this model to rate its performance (a SIR greater than 1 indicates a lower performance).

The common feature (and limitation) of these risk indicators is that they exclusively take into account individual factors to assess SSI risk. Nevertheless, it seems important to consider data hierarchy in order to determine the role played by other specific risk factors, such as ward or hospital characteristics. A suitable statistical method for analyzing grouped or clustered data is multilevel modeling, which has the following advantages: correction of standard error underestimation, examination of cross-level interactions, estimation of the coefficient variability at group level, and analysis of contextual effects after adjusting for individual variables, while also accounting for the non independence of within-group observations [15]–[17].

The aim of this study was, therefore, to improve the predictive performance of an individual-based SSI risk model by investigating the adequacy of a multilevel hierarchical structure.

## Methods

### Data Collection

The RAISIN active surveillance system has been fully described elsewhere [18]. Every year, volunteer surgical wards are asked to complete a survey over a three-month period chosen at their own discretion between January 1, and June 30. In each participating facility, all consecutive surgery procedures occurring during the first 2 months are included with the exception of reoperations due to SSI. Then, a 30 day follow-up is theoretically organized after the procedure (including post hospital discharge) and stopped in case of SSI occurrence. To avoid reporting bias, a standardized questionnaire is completed for each patient by the surgical team with the help of an infection control practitioner to document peri- and post-operative data [19]. An SSI diagnosis is made according to standardized CDC criteria. Every type of surgery is taken into account, including ambulatory surgery (provided a follow-up is possible). Quality control is supervised by the local Coordination Centre for Nosocomial Infection Control (CCLIN). At national level, the database is maintained by the 5 CCLINs involved in the RAISIN surveillance system, in cooperation with the French Institute for Public Health Surveillance.

The 2011 national SSI data consisted of 330202 patients from 2433 wards and 756 hospitals (27.9% of all French hospitals). Among these participating facilities, 50.8% were private hospitals, 8.2% were semi-private hospitals and 41.0% were public hospitals.

**Figure 1:**
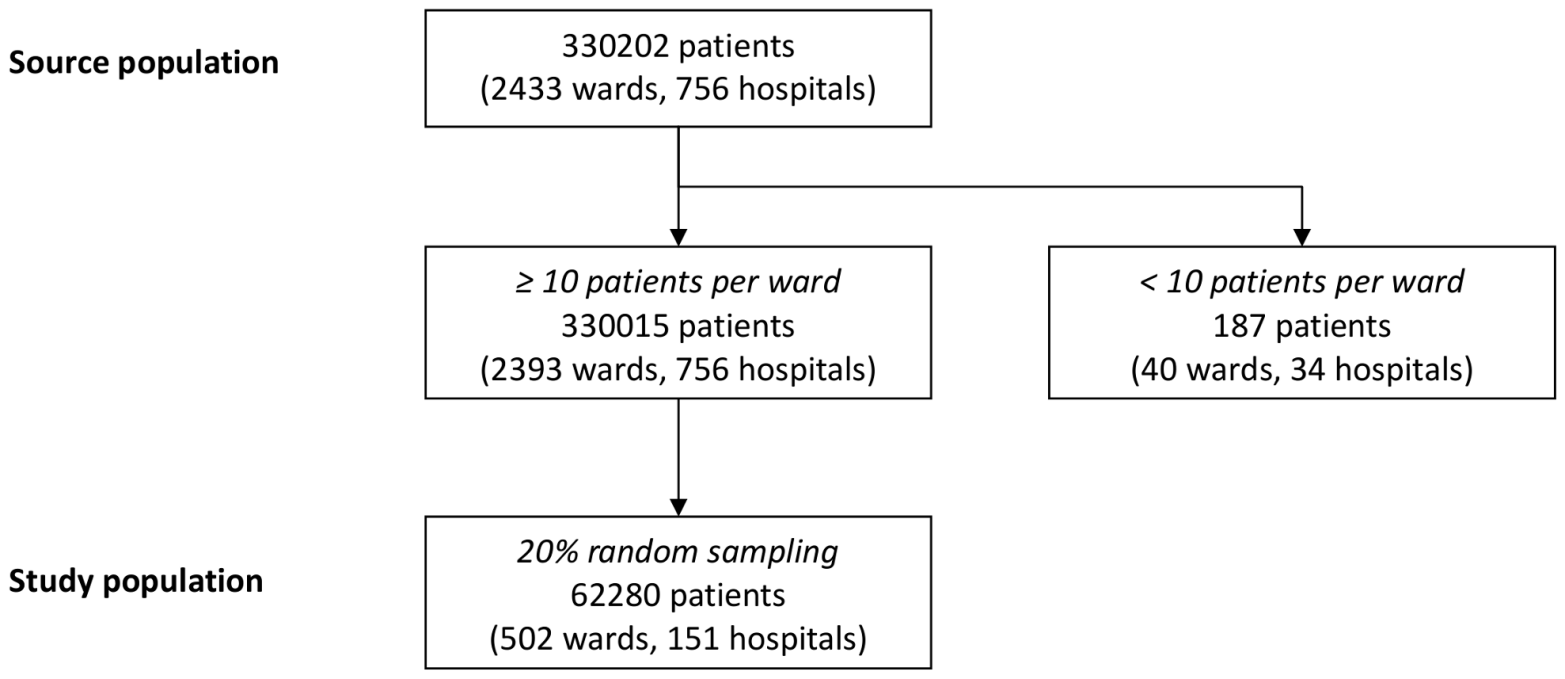
Flow diagram for the surgical site infection study (French RAISIN surveillance system, 2011).

To generate unbiased and accurate estimates from multilevel models, we limited our study to wards of at least 10 patients. We randomly selected 20% of the hospitals from within this subset, thus providing a study population of 151 hospitals, 502 wards and 62280 patients (Figure 1).

To assess the added-value of a multilevel model as opposed to conventional single level models, the following patient characteristics were considered: gender (male vs. female), age (<65 years vs. ≥65 years), ASA physical status score [20], duration of preoperative hospitalization (<48 hours vs. ≥48 hours), wound class according to the Altemeier classification, endoscopic surgery (yes vs. no), type of surgical procedure (gastrointestinal, gynecologic, cardiovascular, orthopedic, ophthalmic, others), duration of surgery (≤75^th^ percentiles vs. >75^th^ percentiles), emergency status of surgery (yes vs. no), ambulatory surgery (yes vs. no), duration of post surgery follow-up (<15 days vs. ≥15 days), time of SSI occurrence (during hospitalization vs. after discharge), depth of infection (superficial, deep, organ/space), mean time between surgery and SSI diagnosis, second surgical procedure (yes vs. no), patient outcome (alive vs. deceased within 30 days). Individual variables with a proportion of missing values greater than 10% were discarded from analyses. No ward-level explanatory variable was available. Regarding hospital level, two characteristics were considered: status (public, private, semi-private) and hospital category (private hospital, teaching hospital, district hospital, regional hospital, military healthcare facility, cancer hospital).

### Statistical Analysis

To take into account the hierarchical structure of data, analyses were initially performed using a 3-level (patient, ward, hospital) hierarchical logistic regression model. The binary outcome was the occurrence of an SSI during the first 30 days post-surgery. Three successive multilevel models were fitted to the data. An empty model (model 1) was built with a random intercept at patient, ward and hospital levels containing no independent variables at any level, in order to determine the initial distribution of the variance of the dependent variable between the three levels. Heterogeneity within levels was considered significant at the p value threshold of 0.05. Patient characteristics with a p value <0.20 in a prior univariate multilevel analysis were included in a multivariate multilevel model, allowing for the probability of SSI to vary across wards but assuming that the effects of individual explanatory variables were the same for each ward (model 2 or random intercept model). Furthermore, in the third model (model 3 or random coefficient model), we introduced random coefficients to allow explanatory variable effects to vary between wards. Parameters were estimated using the simulation-based Markov Chain Monte Carlo (MCMC) procedure for discrete response multilevel models in a Bayesian framework [21]. To quantify heterogeneity between wards, the median odds ratio (MOR) was calculated. The aim of this measure is to translate the group level variance in the widely used odds ratio scale, which has an intuitive interpretation, and is statistically independent from the prevalence of the outcome [22]. Ninety-nine percent credibility intervals (CI) for the MOR were calculated using the 2.5^th^ and 97.5^th^ percentiles of the posterior distribution of the ward variance.

The final model was cross-validated in an independent 20% hospital sample, randomly selected from the same 2011 national database after exclusion of the original 151 hospitals from the study population. This validation sample comprised 51348 patients. Coefficients from multilevel modeling performed on the study population were applied to the validation population, as well as the coefficients from a single level logistic regression model, considered as the reference model (also using the 2011 data to allow direct comparison) [9].

Discriminative accuracy of both models was compared by the means of their respective area under the receiver operating characteristic (ROC) curve, using the Hanley and Mc Neil method [23], [24].

Descriptive analyses were performed using the software SAS version 9.3 (SAS Institute, Cary, NC, USA) and multilevel modeling was carried out using the software MLwiN version 2.23 (Centre for Multilevel Modelling, Bristol University, UK).

### Ethics

This study was given ethical clearance by the French data protection authority (n°326452). As only anonymous data were extracted from patients’ medical files for this surveillance study, the French data protection authority deemed that verbal informed consent was acceptable provided patients were supplied with, as was the case, a document describing the objectives of the study, their right to access the data collected concerning them, and their right to have it rectified (in compliance with act n°78–17 of 6 January 1978 on Information Technology, Data Files and Civil Liberties, amended by act n° 2004–801 of 6 August 2004 relating to the protection of individuals with regard to the processing of personal data).

## Results

### Study Population

Patients’ characteristics are reported in Table 1. The study population was mainly female (56.3%) and age <65 years (58.6%). The vast majority of patients were hospitalized less than 48 hours (92.3%) prior to surgical procedure and had an Altemeier wound class ≤2 (93.0%). Follow-up after surgery was ≥15 days for 65.0% patients.

**Table 1:** Demographic and clinical characteristics of patients according to SSI status (62280 patients, French RAISIN surveillance system, 2011).

Characteristics	No. (%) of patients with SSI(*N* = 623)	No. (%) of patients without SSI(*N* = 61657)	Total(*N* = 62280)
Gender			
Male	308 (49.4)	26409 (42.8)	26717 (42.9)
Female	315 (50.6)	34731 (56.4)	35046 (56.3)
Unspecified	0 (0.0)	517 (0.8)	517 (0.8)
Age			
<65 years	367 (58.9)	36146 (58.6)	36513 (58.6)
≥65 years	256 (41.1)	25504 (41.3)	25760 (41.3)
Unspecified	0 (0.0)	7 (0.1)	7 (0.1)
ASA physical status score			
≤2	432 (69.3)	49261 (79.9)	49693 (79.8)
>2	183 (29.4)	10635 (17.2)	10818 (17.4)
Unspecified	8 (1.3)	1761 (2.9)	1769 (2.8)
Duration of preoperative hospitalization			
<48 hours	518 (83.1)	56994 (92.3)	57512 (92.3)
≥48 hours	105 (16.9)	4662 (7.6)	4767 (7.6)
Unspecified	0 (0.0)	1 (0.1)	1 (0.1)
Altemeier wound class			
≤2	531 (85.2)	57407 (93.1)	57938 (93.0)
>2	85 (13.7)	2560 (4.2)	2645 (4.3)
Unspecified	7 (1.1)	1690 (2.7)	1697 (2.7)
Endoscopic surgery			
Yes	129 (20.7)	9920 (16.1)	10049 (16.1)
No	487 (78.2)	49761 (80.7)	50248 (80.7)
Unspecified	7 (1.1)	1976 (3.2)	1983 (3.2)
Type of surgical procedure			
Gastrointestinal	224 (36.0)	12385 (20.1)	12609 (20.2)
Gynecologic	138 (22.1)	10195 (16.5)	10333 (16.6)
Cardiovascular	46 (7.4)	3762 (6.1)	3808 (6.1)
Orthopedic	63 (10.1)	15620 (25.3)	15683 (25.3)
Ophthalmic	7 (1.1)	7438 (12.0)	7445 (11.9)
Others	137 (22.0)	12053 (19.5)	12190 (19.6)
Unspecified	8 (1.3)	204 (0.3)	212 (0.3)
Duration of surgery			
≤75^th^ percentile	440 (70.6)	51371 (83.3)	51811 (83.2)
>75^th^ percentile	183 (29.4)	10286 (16.7)	10469 (16.8)
Emergency status of surgery			
Yes	115 (18.5)	6894 (11.2)	7009 (11.3)
No	499 (80.1)	53439 (86.7)	53938 (86.6)
Unspecified	9 (1.4)	1324 (2.1)	1333 (2.1)
Ambulatory surgery			
Yes	51 (8.2)	17180 (27.9)	17231 (27.7)
No	572 (91.8)	43913 (71.2)	44485 (71.4)
Unspecified	0 (0.0)	564 (0.9)	564 (0.9)
Duration of follow-up			
<15 days	426 (68.4)	21371 (34.7)	21797 (35.0)
≥15 days	197 (31.6)	40286 (65.3)	40483 (65.0)

Of the 62280 patients included, 623 SSI were diagnosed (1%), 40.3% of which were diagnosed during hospitalization. Forty-eight percent of SSI were superficial, 33.4% were deep incisional infections and 16.8% were organ/space infections. The median time between surgery and SSI diagnosis was 10 days, and 35.5% of the patients required a second surgical procedure. Ten patients died during hospitalization. The main statuses of the hospitals were private (50%) or public (46.2%).

### Multilevel Modeling

Results from the empty model revealed a significant heterogeneity of SSI occurrence between individual wards (p<10^−6^), but not between individual hospitals (p  = 0.32). Patient and ward levels were therefore kept in the subsequent analyses. The hospital level was discarded as it did not contribute to variability of SSI occurrence. In the resulting two-level empty model, ward-level variance could be characterized by a MOR of 3.02 (95% CI 2.47 to 4.71).

Multivariate results are presented in Table 2. In model 2, SSI occurrence was significantly higher among males (p  = 0.04), patients with an ASA score >2 (p<10^−8^), patients with a duration of preoperative hospitalization ≥48 hours (p<10^−3^), patients whose surgical wound was contaminated or dirty according to the Altemeier wound class (p<10^−6^), and patients whose surgery duration was greater than the 75^th^ percentile value for similar procedures (p<10^−9^). In contrast, SSI occurrence was lower among patients who underwent endoscopic surgery (p<10^−2^); patients who underwent orthopedic surgery (p<10^−6^), ophthalmic surgery (p<10^−2^), or other types of surgery (p  = 0.002) (versus gastrointestinal surgery); patients who underwent ambulatory surgery (p<10–8); patients with a follow-up≥15 days after the operation (p<10^−9^). When including these patient-level variables, ward-level variance remained significantly different from zero (p<10^−9^) with a MOR of 3.59 (95% CI 3.03 to 4.33).

**Table 2:** Results of the multilevel logistic regression models (62280 patients, French RAISIN surveillance system, 2011).

Variable	Model 1	Model 2 OR (95% CI)	Model 3 OR (95% CI)
Female gender		0.80 (0.65–0.99)	0.83 (0.67 to 1.04)
Age ≥65 years		1.14 (0.94 to 1.39)	1.15 (0.93 to 1.41)
ASA score >2		2.03 (1.62 to 2.53)	1.99 (1.58 to 2.51)
Duration of preoperative hospitalization ≥48 hours		1.62 (1.27 to 2.08)	1.63 (1.26 to 2.11)
Altemeier wound class >2		2.19 (1.62 to 2.95)	2.09 (1.55 to 2.81)
Endoscopic surgery		0.69 (0.54 to 0.89)	0.70 (0.53 to 0.92)
Type of surgical procedure			
Gastrointestinal		reference	reference
Gynecologic		0.74 (0.49 to 1.13)	0.94 (0.60 to 1.47)
Cardiovascular		0.58 (0.34 to 1.00)	0.59 (0.31 to 1.11)
Orthopedic		0.30 (0.19 to 0.46)	0.31 (0.18 to 0.50)
Ophthalmic		0.07 (0.02 to 0.19)	0.06 (0.02 to 0.19)
Others		0.49 (0.33 to 0.71)	0.52 (0.35 to 0.76)
Duration of surgery >75^th^ percentile		2.12 (1.73 to 2.59)	2.11 (1.71 to 2.62)
Emergency status of surgery		0.96 (0.74 to 1.23)	0.95 (0.73 to 1.23)
Ambulatory surgery		0.36 (0.25 to 0.51)	0.35 (0.24 to 0.50)
Duration of follow-up≥15 days		0.10 (0.08 to 0.12)	0.19 (0.12 to 0.30)
MOR (95% CI)	3.02 (2.47 to 3.71)	3.59 (3.03 to 4.33)	Follow-up<15 days: 6.92 (5.31 to 9.07)Follow-up≥15 days: 3.32 (2.49 to 4.84)

OR, odds ratio; CI, credibility interval; MOR, median odds ratio; model 1: empty model; model 2: two-level random intercept model; model 3: two-level random coefficient model.

The final model (model 3) was as follows:
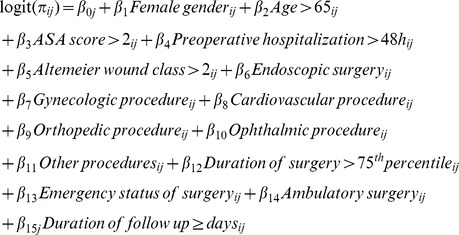



with:

*π_ij_*: the probability of SSI occurrence in patient *i* in ward *j.*

*i*: 1.number of patients.

*j*: 1.number of wards.
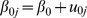


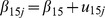



*u*_0*j*_: random intercept effect.

*u*_15*j*_: random coefficient effect.

Corresponding exponentiated estimates (odds ratios) are reported in Table 2. The random coefficient for follow-up duration (*u_15j_*) was statistically significant (p<10^−9^), while the conclusions regarding the effects of patient variables were unchanged. For a duration of follow-up<15 days, the MOR was 6.92 (95% CI 5.31 to 9.07), whereas for a follow-up≥15 days, the MOR was 3.32 (95% CI 2.49 to 4.84). No significant random effect was highlighted for the remaining patient-level variables.

### Validation

**Figure 2:**
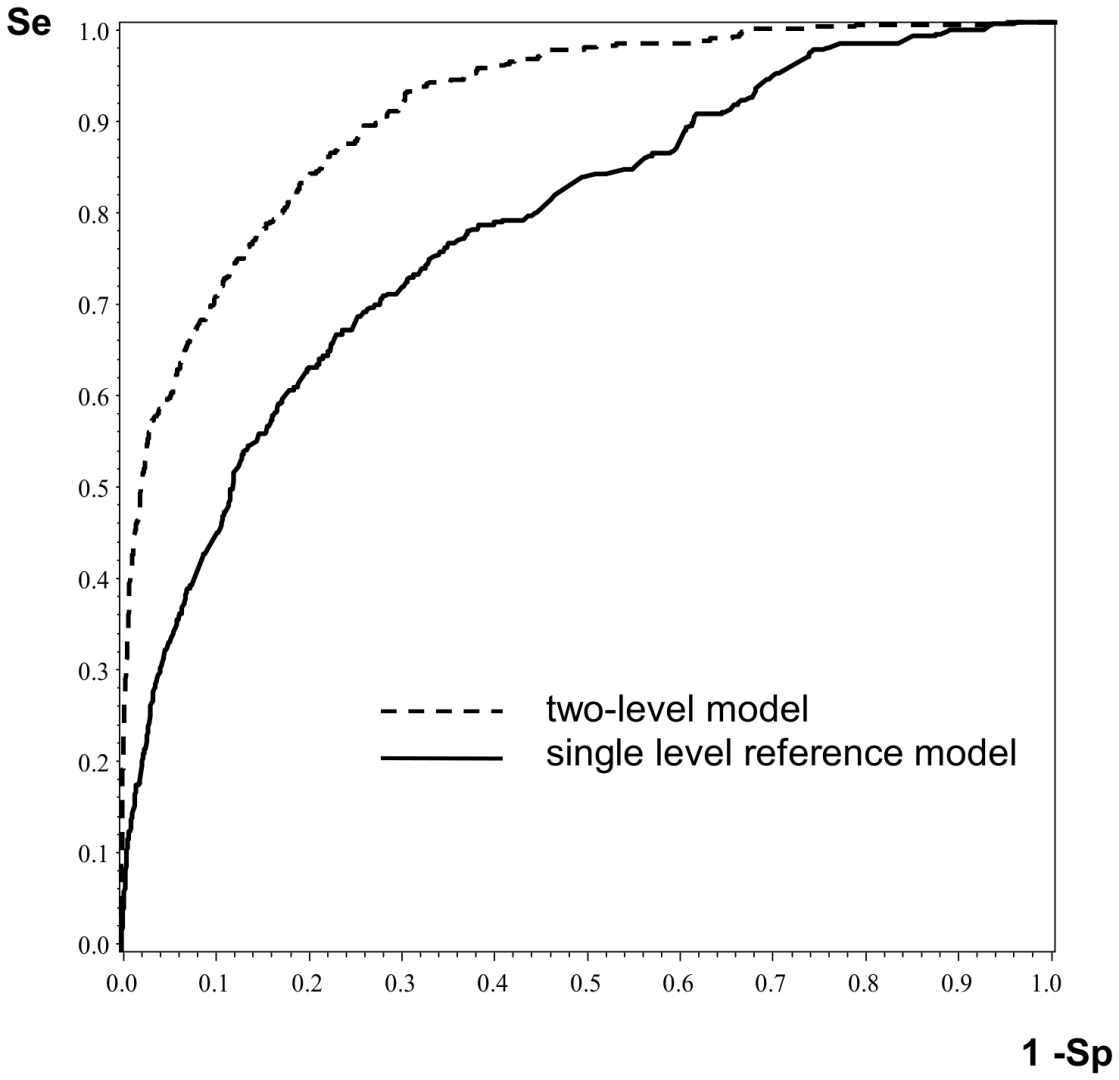
ROC curves for multilevel logistic regression model and single level reference logistic regression model (French RAISIN surveillance system, 2011).

ROC curves for the multilevel logistic regression model and the reference single level logistic regression model (both for 2011) are reported in Figure 2. The risk factors taken into account are the same in both models. However, variables in the reference single level model have no subscript j that would indicate to which ward the patients belong (and therefore no random effects are estimated). When exclusively taking into account individual factors in the reference model, the area under the ROC curve was 0.73. The multilevel logistic model (considering ward variability) significantly improved the discriminative accuracy (p<10^−9^), with an area under the ROC curve of 0.84.

## Discussion

This study sheds new light on the respective contribution of patient-, ward- and hospital-levels to SSI occurrence and demonstrates a significant impact of the ward level in itself (i.e. with no ward-level explanatory variable) after adjusting for patient-level variables.

The strengths of the study stem from a large population base, a high completeness rate, and a sound statistical methodology. To avoid convergence problems, we randomly selected a 20% sample from the 2011 database, comprising a significant number of wards (502) and a sufficient number of patients per ward (≥ 10 patients). These criteria are recognized as crucial for valid and reliable estimates of the fixed and random effects from multilevel analyses [25], [26]. Successive controls at local, regional and national level resulted in good quality data in terms of completeness (less than 1% missing data) and consistency. Thus we were able to use a large number of the variables available in the French RAISIN national database. Assuming that the organization or activity of a ward or hospital might have an impact above individual characteristics, we used multilevel modeling techniques with the distribution-free MCMC method, to take into account the hierarchical structure of the data, the non-independence of within-group observations, and to obtain better parameters estimation.

There are a few limitations to this study that should be noted. First, as involvement in the surveillance system is voluntary, wards paying more attention to infection prevention measures may be overly-represented in the database. Nevertheless, this should not affect our main results as we have highlighted the added-value of a two-level model compared to a single level reference model, all else being equal. Second, lack of information regarding some risk factors difficult to collect in a routine surveillance system (preoperative skin preparation, antibiotic prophylaxis, hand hygiene compliance) may be the source of potential residual confounding. Third, the completeness of the follow-up is questionable as inferred by the proportion of patients without SSI and with a follow-up<15 days (34.7%, Table 1), illustrating an insufficient compliance with the surveillance requirements. However, as SSI surveillance policy is implemented at the ward level in the RAISIN system, any difference in follow-up completeness is for the most part accounted for by the between-ward heterogeneity. Fourth, a 30 day follow-up is quite a short period of time considering SSI may occur some months or years after surgery (especially in the case of surgical implant). Lastly, between-ward heterogeneity was revealed but we were unable to further explore ward-level factors potentially explaining this variability. In this respect, the variable “ward’s post-hospitalization follow-up procedure” (optional in the surveillance network) could not be introduced as a ward-level risk factor because of the excessive amount of missing data (probably in relation with the above-mentioned follow-up completeness issue).

This multilevel study highlights individual factors (patient history, surgical procedure and hospitalization characteristics) as significant risk factors for SSI occurrence, in line with previous studies [9], [18]. It confirms the relevance of collecting these individual data in national surveillance systems such as the surveillance system established by the CDC in the United States (the effectiveness of which has been proven) [4] and at European level by the European Centre for Disease Prevention and Control (ECDC) [27]. It goes, however, one step further by providing a case-mix adjusted model allowing proper between-ward comparisons as individual risk factors (notably the Altemeier classification) are accounted for.

In an empty model, we found a between-patient and between-ward heterogeneity. Conversely, hospital-level had no significant effect on SSI risk. This result emphasizes the importance of taking into account the organizational context in which care is delivered to predict the SSI risk [28], both from a clinical and statistical point of view. This approach is consistent with recent studies that have reported the benefit of simultaneously including both individual and aggregated characteristics in health sciences, particularly in the context of infectious health outcomes [29]–[35]. In the multivariate multilevel model 2, the MOR of 3.59 (95% CI, 3.03–4.33) shows that, in the median case, the residual heterogeneity between wards increases by 3.59 times the individual odds of SSI when randomly picking out two persons in different wards. We also found an increased heterogeneity between wards with follow-up<15 days (MOR  = 6.92 [95% CI, 5.31–9.07]). This may reflect a lack of systematic reporting procedure in some wards. As the vast majority (67%) of SSI occur in the first 15 postoperative days [9], reported SSI may subsequently be concentrated in wards that comply with the surveillance requirements, increasing the between-ward heterogeneity during this period.

In conclusion, the present study provides evidence of the existence of ward-level effects over and above risk factors present at patient level (i.e. independently from patient case-mix). As the ward-level significantly improved the discriminative accuracy, a new two-level risk model (possibly stratified by surgical procedure type) should be implemented to allow clinicians both to predict risk in individual patients and to have at their disposal a better estimate of the expected number of SSI in their respective wards. From a pragmatic point of view, the ward housing a given patient is an easy-to-collect and reliable data element, making the two-level risk model applicable to other settings. Some external validation should, however, be conducted to confirm the robustness of the results stated above. Further work is also required to establish the potential added-value of ward characteristics (post-discharge follow-up process, preoperative skin preparation, hand hygiene compliance, antibiotic prophylaxis policy) to be collected systematically.
